# A Multi-Bioassay Integrated Approach to Assess the Antifouling Potential of the Cyanobacterial Metabolites Portoamides

**DOI:** 10.3390/md17020111

**Published:** 2019-02-12

**Authors:** Jorge Antunes, Sandra Pereira, Tiago Ribeiro, Jeffrey E. Plowman, Ancy Thomas, Stefan Clerens, Alexandre Campos, Vitor Vasconcelos, Joana R. Almeida

**Affiliations:** 1 Centro Interdisciplinar de Investigação Marinha e Ambiental (CIIMAR), Universidade do Porto, Terminal de Cruzeiros do Porto de Leixões, Av. General Norton de Matos s/n, 4450-208 Matosinhos, Portugal; jorgetantunes@gmail.com (J.A.); sandra.c.pereira28@gmail.com (S.P.); tiago.amribeiro8@gmail.com (T.R.); acampos@ciimar.up.pt (A.C.); 2 Departamento de Biologia, Faculdade de Ciências, Universidade do Porto, Rua do Campo Alegre, P 4069-007 Porto, Portugal; 3 AgResearch Ltd., 1365 Springs Rd, Lincoln 7674, New Zealand; Jeff.Plowman@agresearch.co.nz (J.E.P.); Ancy.Thomas@agresearch.co.nz (A.T.); Stefan.Clerens@agresearch.co.nz (S.C.); 4 Biomolecular Interaction Centre, University of Canterbury, Christchurch P 8140, New Zealand; 5 Riddet Institute, Massey University, Palmerston North P 4442, New Zealand

**Keywords:** biofouling, antifouling, cyanobacteria, natural products, marine biofilms, mode of action

## Abstract

The cyclic peptides portoamides produced by the cyanobacterium *Phormidium* sp. LEGE 05292 were previously isolated and their ability to condition microcommunities by allelopathic effect was described. These interesting bioactive properties are, however, still underexplored as their biotechnological applications may be vast. This study aims to investigate the antifouling potential of portoamides, given that a challenge in the search for new environmentally friendly antifouling products is to find non-toxic natural alternatives with the ability to prevent colonization of different biofouling species, from bacteria to macroinvertebrates. A multi-bioassay approach was applied to assess portoamides antifouling properties, marine ecotoxicity and molecular mode of action. Results showed high effectiveness in the prevention of mussel larvae settlement (EC_50_ = 3.16 µM), and also bioactivity towards growth and biofilm disruption of marine biofouling bacterial strains, while not showing toxicity towards both target and non-target species. Antifouling molecular targets in mussel larvae include energy metabolism modifications (failure in proton-transporting ATPases activity), structural alterations of the gills and protein and gene regulatory mechanisms. Overall, portoamides reveal a broad-spectrum bioactivity towards diverse biofouling species, including a non-toxic and reversible effect towards mussel larvae, showing potential to be incorporated as an active ingredient in antifouling coatings.

## 1. Introduction

As soon as a surface is immersed in water, it is rapidly colonized by a wide diversity of organisms in different developmental stages in a process known as marine biofouling [1,2]. Initially, the surface adhesion of organic molecules takes place, followed by the rapid settlement of microorganisms like marine bacteria, protozoans and microalgae [3]. These microfouling communities develop into complex marine biofilms, which provide favourable conditions in which spores of macroalgae and larvae of invertebrates can establish themselves, leading to the attachment of these macrofouling communities [4,5,6]. Marine biofouling is widely known to cause serious problems for marine industries worldwide and it is considered to be one of the most important issues facing marine technology today [7]. Marine fouling results in an increase of fuel consumption of ships and represents a major vehicle for the spread of invasive marine species [8,9]. Macrofouling organisms such as invertebrates and algae are responsible for a significant percentage of marine fouling biomass and for the diminished hydrodynamic performance of a ship [10]. However, marine biofilms that attach to artificial surfaces are also recognized to be a significant issue for a wide range of submerged engineered structures and are known to have a significant role in the corrosion of surfaces immersed in seawater [11].

The most commonly used strategy to prevent marine biofouling is based on antifouling (AF) paints containing active compounds [12]. However, due to their toxicity towards non-target organisms, some AF compounds have raised many environmental issues [13]. After years of application of toxic antifoulants to control biofouling, the International Maritime Organization (IMO) banned the use of organotin compounds in 2008, making urgent the development of environmentally friendly fouling-resistant coatings [14]. Therefore, large investments are applied yearly in the cleaning and the prevention of biofouling species settlement by using biocide-based antifoulants [15,16]. Marine organisms are a promising rich source of compounds for AF, antibiofilm and antipathogenic purposes, which despite increased research interest in recent years remain relatively underexplored [17,18]. Although there was intensive research in the last few years focusing on natural products showing broad and comprehensive AF properties, most of the discovered compounds have a very specific target and bioactivity towards only one or similar species of the marine fouling community [19,20,21]. In the context of natural products biodiscovery, cyanobacteria are a particularly encouraging source of new natural compounds, as they produce a wide range of secondary metabolites with recognized activity in several different biological responses [22,23], including effective and less toxic alternatives to combat marine biofouling, which might replace the biocide-based coatings currently in use [18]. However, being of natural origin does not guarantee that the AF compounds are non-toxic, and the ideal AF compound should be deterring rather than toxic to the target biofouling species [12]. As recently put forward by the Biocidal Products Regulation (BPR) of the European Union (528/2012), studies to elucidate AF mechanisms are considered to be a prerequisite for the registration and commercialization of any new antifoulant. It is thus of major importance to understand the molecular targets of newly discovered AF compounds because this will also help to identify the molecules or pathways involved in the settlement of biofouling organisms [21], and understand if alterations of these pathways are reversible after exposure. Furthermore, despite the importance of marine biofilms in early stages of biofouling formation [1,2], only a few natural compounds have been isolated with the capacity to disrupt marine biofilms [16,22]. Consequently, more chemical studies and broad-sprectrum screening are needed to identify natural products that are active against different targets [23,24].

Portoamides are cyclic dodecapeptides previously isolated in our group from the cyanobacterium *Phormidium* sp. LEGE 05292 from the Blue Biotechnology and Ecotoxicology Culture Collection (LEGE CC, http://www.ciimar.up.pt/legecc/), based on their allelopathic effect on the microalga *Chlorella vulgaris* [25]. Portoamides A and B in natural mixtures also showed synergistic allelophatic activity towards other co-occuring microalgae (*Ankistrodesmus falcatus* and *Chlamydomonas reinhardtii*) and cyanobacteria (*Cylindrospermopsis raciborskii*) species [25]. Based on these previously recognized properties of inhibiting the growth of co-occurring microalgae and cyanobacterial species [26], the present work aims to investigate if portoamides are active against diverse marine biofouling organisms to be employed as non-toxic AF agents. The AF potential of portoamides was tested against both micro- and macrofouling species, specifically against relevant marine biofilm-forming bacteria and diatoms and also towards the adhesive larvae of the macrofouling mussel *Mytilus galloprovincialis*. In addition, the AF molecular targets of these metabolites were studied to determine their mode of action against *Mytilus galloprovincialis* larvae, and their potential marine ecotoxicity was also evaluated. In this study, this multi-bioassay approach will allow for determination of whether portoamides are effective as broad-spectrum and eco-friendly AF compounds and if they follow the new guidelines for the discovery of “clean” natural AF agents.

## 2. Results and Discussion

### 2.1. Mytilus galloprovincialis Larvae Antisettlement Activity and Recovery

**Figure 1:**
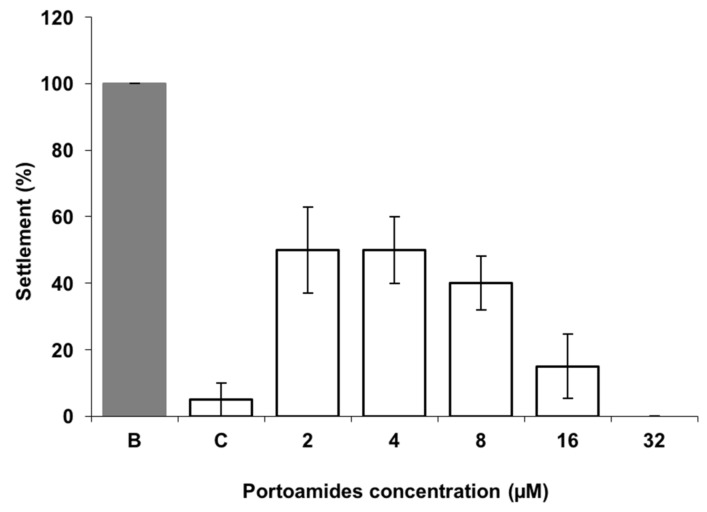
Dose–response antisettlement activity of portoamides towards plantigrade larvae of the mussel *Mytilus galloprovincialis.* B: DMSO control (0.01%); C: 5 µM CuSO_4_ as the positive control.

Portoamides demonstrated a highly significant activity towards the settlement of the mussel *Mytilus galloprovincialis* plantigrades larvae for all the concentrations tested (*p* < 0.01) when compared to the negative control, resulting in a complete inhibition of the larval settlement at a concentration of 32 µM, and an 50% response concentration (EC_50_) value of 3.16 µM/4.86 μg·mL^−1^ (Figure 1).

**Figure 2:**
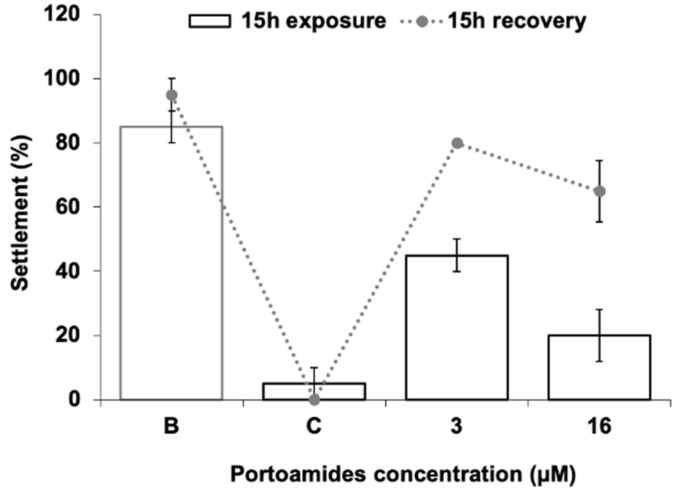
Settlement response of plantigrade larvae of the mussel *Mytilus galloprovincialis* after 15 h of exposure to portoamides followed by 15 h of recovery in filtered seawater. B: DMSO control (0.01%); C: 5 µM CuSO_4_ as the positive control.

This antisettlement bioactivity is highly relevant as it is well below the reference value for EC_50_ (<25 μg·mL^−1^) established by the U.S. Navy program for suitable AF compounds. This EC_50_ value is lower than that of some previous AF compounds isolated from marine organisms [27,28,29], and their effectiveness is within the concentration range of synthetic nature inspired AF compounds tested in the same experimental conditions using *M*. *galloprovincialis* plantigrades antisettlement responses [30,31]. Considering pure compounds isolated from cyanobacterial strains and tested for antisettlement activity, portoamides are more effective than hantupeptin C (EC_50_ = 10.6 μg·mL^−1^), but less potent than isomalyngamide A (EC_50_ = 2.6 μg·mL^−1^), majusculamide A (EC_50_ = 0.54 μg·mL^−1^), and dolastatin 16 (EC_50_ = 0.003 μg·mL^−1^), all isolated from the cyanobacterium *Lyngbya majuscula* [32]. However, these compounds were only tested against *Balanus amphitrite* cyprids and larval sensitivity among invertebrate species might be distinct. Additionally, dolastatin 16 is very potent against cyprids settlement, but also toxic at low concentrations (LC_50_ = 20 μg·mL^−1^). The same was found for the compound Maculalactone A isolated from the cyanobacterium *Kyrtuthrix maculans*, which showed AF potential in field tests, but was found to exert toxicity (LC_50_ = 4.2 μg·mL^−1^) to *B. amphitrite* larvae [33]. The portoamides level of effectiveness towards *M*. *galloprovincialis* larvae is also higher than that of the commercial agent ECONEA^®^, which has a reported EC_50_ value of 4.01 µM [30]. Despite the high effectiveness in preventing *M*. *galloprovincialis* plantigrades settlement, portoamides caused no mortality to the target species at any of the concentrations tested, while the commercial AF agent ECONEA^®^ was found to exert some toxicity (LC_50_ = 107.89 μM·mL^−1^) [31]. The lack of toxicity of portoamides towards mussel larvae at the tested concentrations suggests that portoamides have a deterring effect rather than a toxic mechanism towards the mussel larvae. In addition, the settlement recovery bioassay pointed out that after a recovery period of 15 h in fresh seawater, larval settlement responses increased by 35% and 45%, when compared to the responses immediately after portoamides exposure (3 µM and 16 µM, respectively), being also not significantly different from the negative control (Figure 2). This might indicate that the mechanisms involved in larval antisettlement are reversible at least after acute exposure to portoamides.

This implies that portoamides have a potential to be employed as environmentally friendly AF agents against mussel attachment prevention, which is environmentally relevant, as *Mytilus* sp. are one of most prevalent macrofouling organisms worldwide, representing a significant part of the biofouling biomass [34,35].

### 2.2. Antibacterial and Antimicroalgal Activities

**Figure 3:**
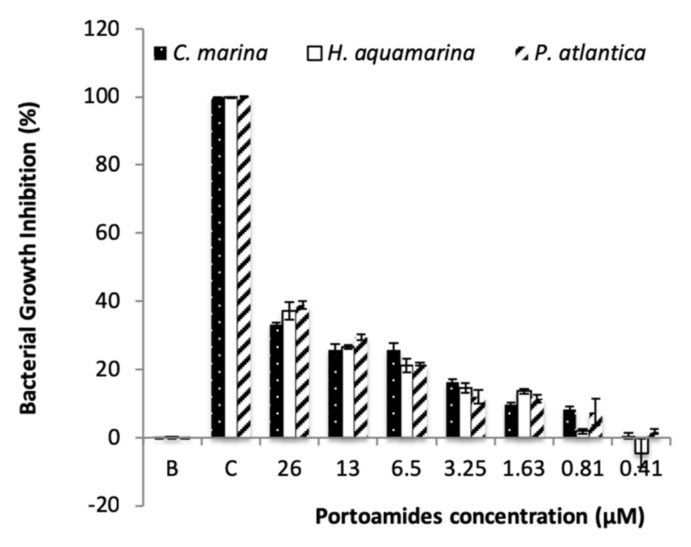
Antibacterial activity of portoamides towards five biofilm-forming marine bacteria *Cobetia marina*, *Pseudoalteromonas atlantica* and *Halomonas aquamarina*. B: 0.1% DMSO; C: 1:100 dilution of penicilin-streptomycin-neomycin stabilized solution (Sigma P4083).

The capacity of portoamides to interfere with microfouling growth was assessed by screening portoamides against a panel of marine bacteria and microalgae species involved in marine biofilm formation. In total, portoamide bioactivity was tested against five marine bacteria and four microalgae strains. The results showed significant activity of portoamides against three of the tested marine bacteria, *Cobetia marina* (inhibition of 23.3%), *Halomonas aquamarina* (inhibition of 21.0%) and *Pseudoalteromonas atlantica* (inhibition of 21.5%) at a concentration of 6.5 µM, but no significant inhibitory activity against *Vibrio harveyi* or *Roseobacter litoralis* was detected. In the dose–response assay, portoamides displayed an inhibitory activity of 33.8% against *C*. *marina*, 37.1% against *H*. *aquamarina* and 38.9% against *P*. *atlantica* at the highest tested concentration of 26 µM (Figure 3).

In this study, the reported EC_30_ value of portoamides against *Halomonas aquamarina* (Table 1) was similar to the activity of sphaerocyclamide, a cyanobactin isolated from a *Sphaerospermopsis* sp. strain [36], and also in line with the activity of nature inspired sulfated compounds [30], which were tested against the same bacterial strain.

**Figure 4:**
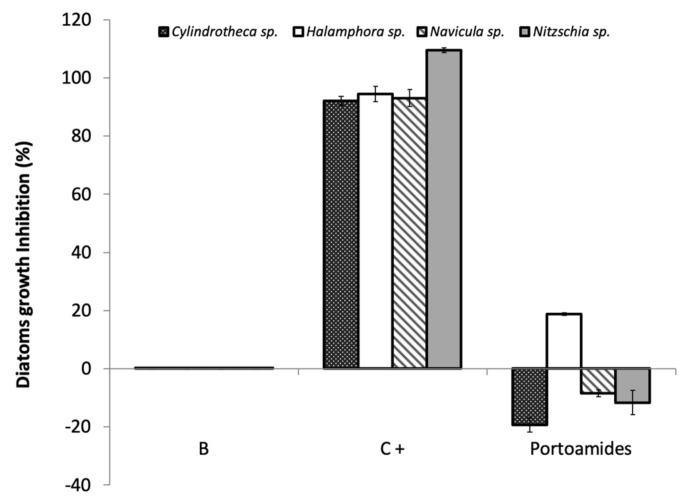
Antimicroalgal activity of portoamides at a concentration of 6.5 µM towards four biofilm-forming marine diatoms *Cylindrotheca* sp., *Halamphora* sp., *Nitzschia* sp. and *Navicula* sp. (B: 0.1% DMSO); 3.55 µM cycloheximide was used as the positive control (C+).

Interestingly, the EC_30_ value of portoamides is lower than the EC_30_ value of the commercial product ECONEA® (EC_30_ = 15.31 µM) [31]. Portoamides bioactivity is also similar or stronger than other natural AF compounds tested against other *H*. *aquamarina* strains [37,38]. Although the activity of portoamides against *C*. *marina* is lower when compared to some reported natural AF compounds or crudes [39,40], portoamides were also more efficient in inhibiting the growth of *C*. *marina* than ECONEA®. Portoamides activity against *P*. *atlantica* was significanly higher than that of other natural products [35], some of which such as eleganediol and eleganolone had EC_50_ values in the order of 300 to 400 µM [41], while the portoamides-extrapolated EC_50_ value is 55.43 µM. Portoamides have already shown a significant inhibitory activity against the microalgae *Chlorella vulgaris* (IC_50_ =12.8 µg·ml^−1^) [25], towards the co-occurring microalga *Ankistrodesmus falcatus* (IC_50_ = 24.7 µg·ml^−1^) and *Chlamydomonas reinhardtii* (IC_50_ = 12.6 µg·ml^−1^), and were also bioactive against a strain of the cyanobacterium *Cylindrospermopsis raciborskii* (IC_50_ = 28.4 µg·ml^−1^) species [25]. Those previous results have suggested that portoamides are strongly bioactive secondary metabolites and their underexploited potential was here confirmed by their inhibitory activity towards the growth of several biofilm-forming bacterial strains. No significant inhibitory activity of portoamides was found against diatom growth, except for a slight inhibition of *Halomphora* sp. growth (<20%) at a concentration of 6.5 µM (Figure 4).

Diatoms represent a class of particularly relevant microorganisms in biofouling, which attach to artificial surfaces after the bacterial colonization, forming resilient slimy layers on marine surfaces, and are known to be a difficult group to target with any AF strategy [42]. The low inhibitory effect towards the tested microalgae species suggests that the mechanism responsible for the bactericidal activity of the portoamides does not affect these eukaryotic microorganisms.

### 2.3. Antibiofilm Activity

**Figure 5:**
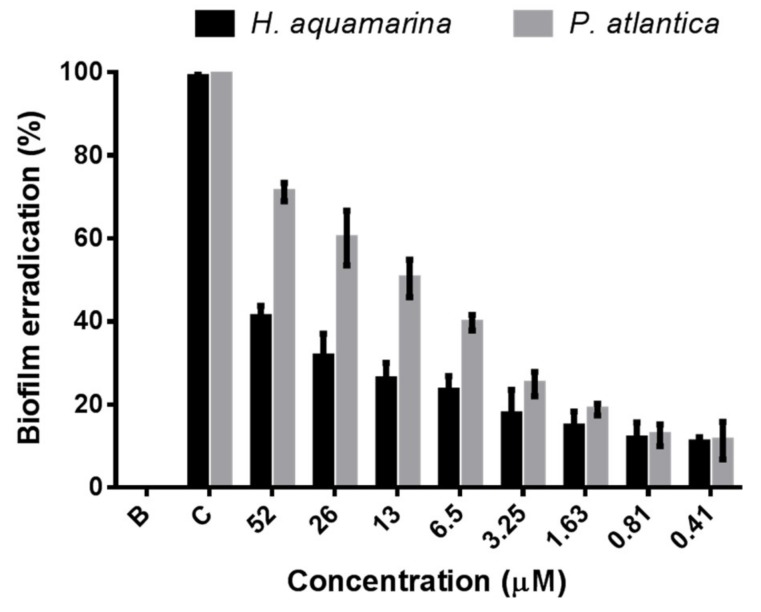
Antibiofilm dose–response activity of portoamides towards the marine bacteria *Halomonas aquamarina* and *Pseudoalteromonas atlantica*. B: 0.1% DMSO; C: 4:100 dilution of penicilin-streptomycin-neomycin stabilized solution (Sigma P4083).

The antibiofilm screening assay showed a higher activity of portoamides against the same bacterial strains in bacterial growth. Portoamides disrupted the biofilm cells of C. marina (10.2% of inhibition), H. aquamarina (25.1% of inhibition) and P. atlantica (44.5% of inhibition), but no significant inhibitory activity was found against either V. harveyi and R. litoralis biofilms at the tested concentration of 6.5 μM. The bacterial strains for which the most significant biofilm disruption was observed were selected to be further tested at higher and lower concentrations of portoamides (52, 26, 13, 6.5, 3.25,1.63, 0.81 and 0.41 μM). The concentration–response assay displayed a strong antibiofilm activity of portoamides against P. atlantica from the lowest concentration of 0.41 μM (11.3% of growth inhibition) to the highest concentration of 52 μM (71.5% of growth inhibition) (Figure 5), when compared to the negative control (EC_50_ = 13.54 µM; EC_30_ = 3.57 µM). 

Similarly, a significant inhibitory activity of portoamides was observed toward H. aquamarina biofilms from the lowest concentration of 0.41 μM (10.9% of growth inhibition) to the highest concentration of 52 μM (41.0% of growth inhibition) (Figure 5), when compared to the negative control (EC_30_ = 17.93 µM), but no relevant disruption of C. marina biofilm was found (<10% of inhibition). Taking into consideration this antibiofilm activity of portoamides and their inhibitory activity against bacterial growth, it can be hypothesized that these natural compounds have a broad activity against those biofilm bacterial strains at different stages of their life cycle, both at the planktonic stage and at the biofilm stage.

The Halomonas genus and Halomonas aquamarina species, in particular, are one of the most common microorganisms constituting marine biofilms, and are particularly prevalent on biofilms attached to the hulls of ships [43,44]. Pseudoalteromonas is among the most preponderant marine bacterial genera recovered from different marine biofilm situations [45,46,47,48]. Compounds isolated from a Mediterranean brown alga were found to be disruptive of a Pseudoalteromonas strain D41, and among the tested compounds, the one with highest activity (dictyol C) had a reported EC_50_ value of 30 μM [41], which is significantly higher than the EC_50_ obtained for portoamides (EC_50_ = 13.54 μM). The activity of these compounds refers to the detachment of already adhered bacteria and not to the inhibition of planktonic growth.

It is known that inhibitors of biofilm formation can serve as potentially efficient antifoulants, and thus biofilm inhibition and quorum-sensing (QS) inhibition bioassays are often used to assess the AF properties of natural compounds [20]. The Calgary Biofilm Device [49] was used to determine the antibiofilm activity of portoamides, and the Minimal Biofilm Eradication Assay (MBEC) was applied to quantify biofilm eradication as a response of compounds bioactivity [49]. Specifically, with the MBEC assay, it is possible to determine both the minimum biofilm eradication concentration (MBEC), simultaneously with the determination of the minimal inhibitory concentration (MIC) of the planktonic growth for a tested compound.

Previous studies which used the MBEC assay reported the disruption of pre-formed biofilms by natural compounds; however, those have focused mostly on pathogenic biofilm strains and not on marine biofilms [50,51]. Other studies have analyzed the response of marine bacteria attachment to surfaces in the presence of natural compounds but have rarely focused on the disruption or on the detachment of already attached biofilms [52], which has importance for AF purposes.

Natural compounds, which can disrupt biofilm formation, may be useful for the biotechnological development of an environmentally friendly protection against marine fouling [53]. There has been a lack of studies quantifying the disruption of marine biofilms, but those assays are essential for future AF studies involving new natural compounds as biofilms constitute the initial stage of marine biofouling [21]. So far, only a few natural products derived from marine microorganisms with antibiofilm activity have been reported [20], and most compounds with antimicrofouling activity have been developed to target microfouling species in their planktonic stage [16]. However, marine biofilm formation allows bacteria to be more resistant to environmental pressures and are the initial stage of the marine biofouling process [2,3]. Therefore, it is promising to exploit natural compounds with excellent antibiofilm activity as potential antifoulants. Portoamides seem to be particularly effective natural compounds in the eradication of biofilms of marine bacterial strains involved in biofouling and surface corrosion. Portoamides also show higher activity than some previously discovered natural products or some AF commercial biocides. This potential should be confirmed in future studies, namely by testing portoamides against other marine biofilm-forming bacterial strains involved in marine fouling situations.

### 2.4. Quorum-Sensing Inhibition (QSI) Activity

**Figure 6:**
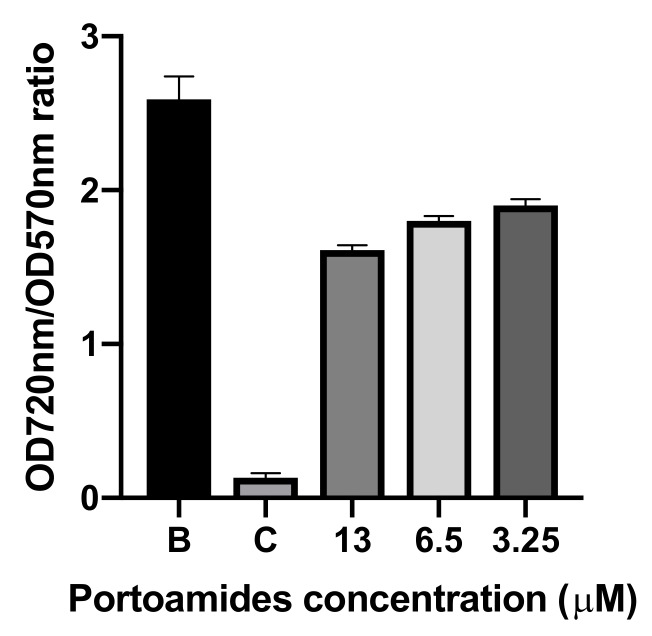
Ratio of the quantification of growth inhibition of *Chromobacterium violaceum* ATCC 12472 (read at 720 nm) when compared to quantification of the production of violacein by the same bacterium (577 nm). B: 0.1% DMSO; C: 1:100 dillution of penicilin-streptomycin-neomycin stabilized solution (Sigma P4083).

The standard QS disk-diffusion assay with Chromobacterium violaceum was used to determine if portoamides interfered with the QS phenomenon while not interfering with bacterial growth which is an ideal strategy to avoid biofilm formation [2,54]. The QS inhibition screening showed that portoamides at 6.5 µM concentration produced a transparent halo in the Chromobacterium violaceum agar cultures, which is associated with the cellular death of the QS C. violaceum reporter strain. The subsequent assay that quantified the inhibition of growth of the reporter strain, when compared to its production of violacein, further demonstrated a significant inhibition of C. violaceum growth by portoamides (31% of growth inhibition at a concentration of 6.5 µM, and 42% of growth inhibition at a concentration of 13 µM; data not shown) with no significant inhibition violacein production (Figure 6).

Portoamides inhibitory activity against C. violaceum (a Gram-negative bacteria) further demonstrates the bactericidal/inhibitory activity of these cyanobacterial natural compounds, while not displaying any detectable QS inhibitory activity. Therefore, portoamides do not appear to have potential as quorum-quenching compounds as they were not shown to interfere with AHL-mediated cellular communication in the conducted assays.

### 2.5. Ecotoxicity Assessment

**Figure 7:**
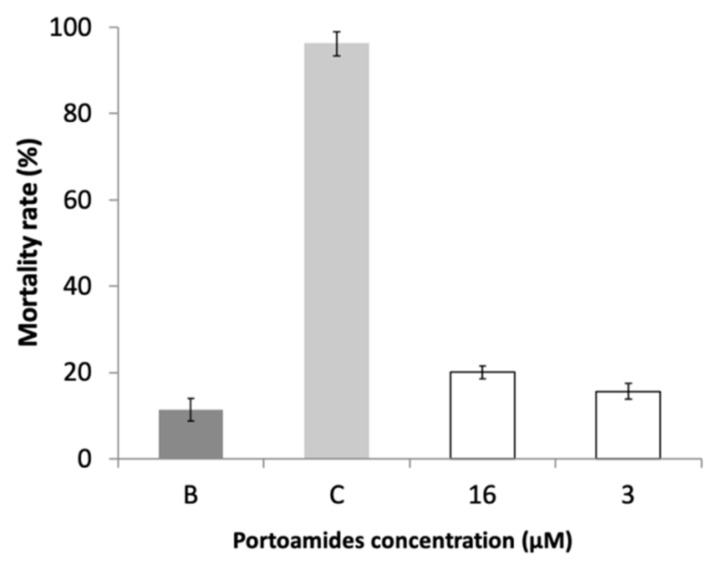
Mortality rate of *Artemia salina* nauplii after 48 h of exposure to portoamides. B: 1% DMSO in filtered seawater. C: K_2_Cr_2_O_7_ at a concentration of 13.6 µM.

To evaluate the viability of portoamides as AF agents, its potential ecotoxicity was assessed using the standard *Artemia salina* bioassay. No significant differences in the *Artemia salina* nauplii mortality rate was found for portoamides at both the tested concentrations (16 µM and 3 µM), when compared with the negative control (*p* < 0.01) (Figure 7). The absence of toxicity of portoamides against this model toxicity organism [55] suggests the potential of portoamides as suitable AF agents with no expressive hazardous effect on the marine environment. However, the toxicity of portoamides towards organisms of other trophic levels should also be tested to fully interpret the absence of toxicity of these compounds. 

### 2.6. Mode of Action Assessment

The assessment of AF molecular targets of AF candidates represents an important complementary information in the establishment of new AF agents. The challenge for the selection of promising AF candidates should include a mode of action elucidation in target biofouling species permitting the explaination of a broad-spectrum effect or a specific adhesive effect of non-toxic compounds that do not act as biocidal agents. To reach this, the differential expression of proteins in the proteome of M. galloprovincialis plantigrade larvae in response to the portoamides when compared to control larvae was analyzed by label-free shotgun proteomics. The complete information regarding differential protein abundance and the molecular functions of identified proteins is summarized in Table 2. Data correspond to the expression of proteins from ten M. galloprovincialis whole plantigrade larvae from the antisettlement bioassays per replicate, and the mean of the four replicates was considered. Portoamides exposure caused the alteration of several proteins, being the most representative proteins attributed to energetic metabolism processes and structural characteristics (Table 2). The most relevant under-expressed proteins participate in ATP synthesis processes (proton-transporting ATPase activity), indicating an inhibition of ATPases activity, which may result in increasing mitochondrial proton gradient and a decline in ATP levels. This effect was previously described in the colon carcinoma cell line HT-29, after exposure to portoamides and attributed as a molecular mode of action [56].

A set of more than ten proteins related with structural constituents and functions were also found to be downregulated in exposed larvae, particularly proteins responsible for actin filament binding (muscle contraction) and structural constituents of the cytoskeleton, including ciliary microtubules units (tubulin alpha and beta chains, and radial spoke head proteins). The impairment in muscle contraction might compromise the activity of the larva foot that is the major organ responsible for the production of threads and all the steps of the adhesive metabolism that enables attachment [57]. 

Tubulin isoforms are the units forming microtubules that are the basic structural elements of mussel cilia present in the gills, and radial spoke head proteins are important components of the axoneme that forms the core of cilia. The cilia have the major function of driving water flow through filtration for respiration and feeding along the ventral groove towards the mouth, so failure in the repair and generation of new cilia might be of great importance in the stress condition and health status of mussels [58]. These molecular consequences related to the modulation of ciliary constituents were previously observed in *Mytilus* sp. in response to warm and cold acclimated conditions [59]. Proteins related with regulatory and transcription/translation processes were also found to be modulated by exposure to portoamides (Table 2).

Overall, these molecular alterations in mussel plantigrade larvae exposed to portoamides seem to be related as a drop in ATP production would contribute to a decrease of energetic requirements available for filtration, and thus this contributes to the downregulation of synthesis and repair processes of the gill ciliary epithelia As the observed mode of action seems to be the failure in proton-transporting ATPases activity, it represents a broad-spectrum effect, increasing the relevance of portoamides as an AF agent as it would be effective in a wide range of biofouling species. In addition, considering that the antisettlement activity is reversible in mussel larvae after a recovery period (Figure 2), this seems to indicate that these molecular targets are subjected to chemical and physiological modulation not causing a permanent damage of the mechanisms responsible for the activity. This might be particularly important in the AF scenario, since the AF active ingredients incorporated into paints should act in a repulsive way and target biofouling species should remain able to colonize alternative substrates. This combination of AF effectiveness, low toxicity and mode of action reversibility is the starting point of the development of suitable AF active ingredients. Some marine natural compounds have also been discovered meeting these characteristics, such as the compound polymeric alkylpyridinium salts (poly-APS) isolated from the Mediterranean marine sponge *Haliclona (Rhizoniera) sarai* and their synthethic analogues which act by neurotransmission blockade/modulation through acetylcholinesterase inhibition, combined with surfactant-like properties that decrease surface tension [60,61]. 

The observed AF profile of portoamides in this work suggests that the mode of action is dictated by different mechanisms depending on the target groups, and the combination of functionalities of these natural products may serve as an inspiration for the development of new AF agents.

## 3. Materials and Methods

### 3.1. Portoamides Isolation and Purification

The cyanobacterial strain *Phormidium* sp. LEGE 05292 (LEGE-CC) was cultured and upscaled in the laboratory for the isolation of portoamides A and B, at a proportion of 3:1 (Supplementary Figure S1), as described in [56]. This defined mixture of portoamides, produced naturally, was maintained and used in all the following experiments and hereafter referred to as portoamides.

### 3.2. Antifouling Bioassays

#### 3.2.1. Mussel Larvae Antisettlement Bioassays

Mussel (*Mytilus galloprovincialis*) juveniles (0.5 cm in diameter) were sampled in intertidal pools during low neap tides, at Memória Beach, North Portugal (41°13’59” N; 8°43’28” W) and appropriately transported to the laboratory. Mussel plantigrade larvae (0.5–2 mm) were screened among the mussel aggregates in a binocular magnifier (Olympus SZX2-ILLT, Tokyo, Japan), isolated with filtered seawater and gently washed to remove adhered organic particles immediately before the bioassays. The selected *M. galloprovincialis* plantigrade larvae were further screened for typical explored behaviour before bioassays. The antisettlement bioassays were performed using 5 larvae per replicate in 24-well microplates with 4 replicates per condition during 15 h, at 18 ± 1 °C, in the darkness [30]. Test solutions were prepared in filtered seawater and obtained by serial dilution (2, 4, 8, 16 and 32 µM) of the compounds stock solution (3.65 mM) in DMSO. A negative control (0.01% DMSO) and a positive control (5 μM CuSO_4_) were included in all bioassays. After exposure, the antisettlement activity was determined by the presence/absence of efficiently attached byssal threads produced by each individual larva for all the conditions tested, and the semi-maximum response concentration that inhibited 50% of larval settlement (EC_50_) and the median lethal dose (LC_50_) was determined, if applicable.

#### 3.2.2. Mussel Larvae Settlement Recovery Bioassays

A settlement recovery bioassay was performed to test if the antisettlement effect observed after acute exposure (15 h) to portoamides is reversible after the same period of time in mussel larvae. The initial exposure bioassay was conducted in the same conditions described in Section 3.2.1. Plantigrade larvae were exposed for 15 h to two portoamides concentrations, 3 and 16 µM, corresponding to the mussel larvae EC_50_ concentration and the concentration used for the mode of action determination, respectively. Negative (0.01% DMSO) and positive controls (5 μM CuSO_4_) were included. After exposure, the antisettlement activity was confirmed by the absence of efficiently attached byssal threads produced by each individual larva when compared to the negative control. Then, the same larvae subjected to portoamides exposure were individually transferred to new test plates with fresh filtered seawater. Controls were included. After 15 h, settlement was evaluated again for all the conditions tested.

#### 3.2.3. Antibacterial Bioassays

For antibacterial activity screening, five strains of marine biofilm-forming bacteria *Cobetia marina* CECT 4278, *Vibrio harveyi* CECT 525, *Halomonas aquamarina* CECT 5000, *Pseudoalteromonas atlantica* CECT 570 and *Roseobacter litoralis* CECT 5395 from the Spanish Type Culture Collection (CECT, Valencia, Spain) were inoculated and incubated for 24 h at 26 °C in Marine Broth (Difco, Detroit, MI, USA) at an initial density of 0.1 (OD_600_) in 96-well flat bottom microtiter plates and exposed at the test concentration. Bacterial growth inhibition in the presence of the tested compounds was determined in quadruplicate at 600 nm using a microplate reader (Biotek Synergy HT, Winooski, VT, USA). Marine broths with 0.1% DMSO and 1:100 penicilin-streptomycin-neomycin stabilized solution (P4083, Sigma-Aldrich, St. Louis, MO, USA) were used as negative (B) and positive (C) controls, respectively. Statistically significant differences between treatments and control measurements were calculated using a Student’s *t*-test with a 95% confidence level.

#### 3.2.4. Antimicroalgal Bioassays

For the antimicroalgal screening, four strains of benthic marine diatoms, *Cylindrotheca* sp., *Halomphora* sp., *Nitzschia* sp. and *Navicula* sp. were purchased from the Spanish Collection of Algae (BEA-Banco Español de Algas, Las Palmas, Spain) and inoculated in f/2 medium (Sigma-Aldrich, St. Louis, MO, USA) with silica at an initial concentration of 2–4 × 10^6^ cells per milliliter, and grown in 96-well flat bottom microtiter plates for 10 days at a temperature of 20 °C. Diatoms growth inhibition in the presence of portoamides at 10 µg/mL was determined in quadruplicate, and quantified by the difference between cell densities among treatments, counted using a Neubauer counting chamber. f/2 medium with 0.1% DMSO and cycloheximide (3.55 µM) were the negative and the positive controls, respectively. Statistically significant differences between treatments and control measurements were calculated using the Student’s *t*-test with a 95% confidence level. The antibacterial and antimicroalgal activity of a commercial agent (ECONEA^®^) was also included in the same bioassay conditions (8.7 µM) as a reference AF compound. The compounds which showed significant inhibitory activity in screening assays were selected for further determination of maximal inhibitory concentrations (IC). A serial dilution of the stock solution was used to obtain final concentrations from 13.5 μM to 3 μM.

#### 3.2.5. Biofilm Inhibition Assay

To test the disruption activity of portoamides against marine bacterial biofilms, the MBEC™ (Minimum biofilm eradication concentration) assay system was employed (Innovotech Inc. Edmonton, Canada) following the protocol of Ceri et al. [49]. Marine bacterial biofilm formation was established by adding 200 μL of bacterial inoculum in MB medium (Difco, Detroit, MI, USA) to MBEC plates and incubating them stagnant at 25 °C for 3 days (*Cobetia marina*, *Vibrio harveyi*, *Halomonas aquamarina* and *Pseudoalteromonas atlantica*) or 5 days (*Roseobacter litoralis*). After biofilm formation for each bacterium, the peg lids of each of the MBEC plates were aseptically removed and rinsed twice with sterile PBS. The plate lids with bacterial biofilms were then added to new 96-well flat bottom microtiter plates (Orange Scientific, Braine- L’Alleud, Belgium) with fresh MB medium and incubated overnight at 25 °C for 24 h and exposed to portoamides (concentrations from 0.41 μM to 52 μM). DMSO (0.1%) was used as a negative control, while a 4:100 dilution penicilin-streptomycin-neomycin stabilized solution (P4083, Sigma-Aldrich, St. Louis, MO, USA) was used as a positive control for biofilm disruption.

To determine biofilm disruption, the peg lids of each of the MBEC plates containing bacterial biofilms were washed twice for 1 min with sterile PBS. Afterwards, the lids of the MBEC plates were placed in a new standard 96-well microtiter plate containing 200 μL of sterile of MB medium. The removal of biofilm cells from the pegs of the MBEC plates was achieved by sonication for 5 min at 30 kHz, and vortexing for 30 s. Afterwards, the MBEC lid is replaced with a standard 96-well plate lid and biofilm susceptibility to portoamides was then measured by reading optical density at 600 nm using a microplate reader (Biotek Synergy HT, Winooski VT, USA). Bacterial growth and turbidity in this situation correspond exclusively to biofilm cell survival which allows for the determination of MBEC.

#### 3.2.6. Quorum-Sensing Inhibition Assay

##### Disk-Diffusion Assay

A standard disk-diffusion assay was used to detect antiquorum sensing activity of the compounds by using a biomonitor strain *of Chromobacterium violaceum* (ATCC 12472) following the protocol developed by McLean [62] with slight modifications. This reporter bacterium regulates violacein production of the pigment by *N*-hexanoyl-l-homoserine lactones (C6-HSL) and is inhibited by acylated homoserine lactones (AHLs) analogues and other antagonists. *C*. *violaceum* 12472 was grown in Luria-Bertani (LB) broth with 1.2% agar. Five milliliters of molten LB agar (0.3%, w/v) was inoculated with 50 mL of a culture of the *C*. *violaceum* 12472 grown overnight in LB. The agar-culture solution was immediately poured over the surface of pre-warmed LB agar plates. Twenty microliters of portoamides (6.5 µM) were pipetted on sterile paper discs, which were then placed on the solidified agar. The plates were incubated overnight at 30 °C and examined for violacein production. Quorum-sensing inhibition (QSI) was detected by a colourless, opaque, but viable halo around the discs. Growth inhibition representing antibacterial activity was detected by a transparent zone/growth inhibition.

##### Quorum-Sensing Inhibition (QSI) Quantitative Assay (Dose–Response Violacein Inhibition Assay)

To quantify the inhibition of QS by the portoamides, the QS reporter strain *Chromobacterium violaceum* (CECT 494/ ATCC 12472) was selected following the protocol of Martinelli [63] and Gemiarto [64]. This strain was incubated in LB medium (Difco) with 3.25 µM, 6.5 µM and 13 µM of portoamides in 96-well flat bottom microtiter plates. LB medium with sterilized ultra-pure water was used as a negative control, and a 1:100 penicilin-streptomycin-neomycin stabilized solution (P4083, Sigma-Aldrich, St. Louis, MO, USA) was used as a positive control for the inhibition of cell growth. The cultures were grown overnight (18 h) at 26 °C with a constant shaking of 50 rpm. After the incubation period, the portoamides-treated culture and the controls were used for the determination of OD at 720 nm (to quantify cell density), using a microplate reader (Biotek Synergy HT, VT, USA). Afterwards, the plates were dried for 30 min at 60 °C and violacein was re-solubilized by the addition of 200 μL of DMSO and the OD was read at 577 nm (to quantify violacein production). Mean OD720/ OD 577 ratios of the portoamides cultures were compared with the untreated cultures using an unpaired Student’s *t*-test (*p* = 0.05) and used to assess the degree of violacein inhibition.

### 3.3. Ecotoxicity Bioassay

Portoamides were tested for marine ecotoxicity using the *Artemia salina* nauplii lethality test [30,52]. *A. salina* eggs were allowed to hatch in nutrient-enriched seawater for approximately 48 h at 25 °C. Test concentrations were 25 and 5 µg/mL and test solutions were prepared in filtered seawater. Bioassays were performed in the darkness in 96-well microplates with eight replicates per condition and 15–20 nauplii per well. K_2_Cr_2_O_7_ (13.6 µM) was included as a positive control and 1% DMSO was used as a negative control. The percentage of mortality was assessed at the end of the exposure period.

### 3.4. Antifouling Mode of Action Towards Mytilus Larvae

#### 3.4.1. Sample Preparation

Ten *M. galloprovincialis* whole plantigrade larvae from the antisettlement bioassays were used per replicate (four replicates per condition were used) and incubated in lysis buffer with 2% (w/v) SDS, 100 mM Tris-HCl, 0.1 M DTT and protease inhibitors (Roche, 11697498001, Basel, Switzerland), pH 7.6 for 1 h with mixing (450 rpm). Additional denaturation was achieved by sample heating (95 °C, 3 min). All samples were clarified at 16,000× *g* for 20 min and total protein was estimated based on the absorbance at 280 nm (1 Abs = 1 mg/mL protein) with Nanodrop (Thermo Scientific, Waltham, MA, USA) before storage at −80 °C. Proteins were subsequently digested following filter aided sample preparation (FASP) [65] with modifications. Briefly, protein samples (100 μg) were alkylated with iodoacetamide, incubated with trypsin (Roche, 03708985001) at an enzyme-to protein ratio of 1:50 (*w*/*w*) for 16 h at 37 °C, in 30 kDa nominal molecular weight limit (NMWL) centrifugal filter units (MRCF0R030, Millipore, Billerica, MA, USA). Peptide samples were loaded in detergent removal spin columns (Thermo Scientific) and the manufacturer instructions followed to get remove detergent residues from the samples. The samples were acidified with formic acid and dried in a Centrivap centrifugal concentrator (Labconco, Kansas City, MO, USA). Finally, the samples were resuspended in 50 μL of mixture composed of 0.1% formic acid and 2% acetonitrile.

#### 3.4.2. LC-MS/MS Analysis

LC-MS/MS was performed on a nanoflow Ultimate 3000 UPLC (Dionex) coupled to an Impact HD mass spectrometer equipped with a CaptiveSpray source (Bruker Daltonik, Bremen, Germany). For each sample, 1 µL of the sample was loaded on a C18 PepMap100 nano-Trap column (300 µm, ID × 5 mm, 5 micron 100 Å) at a flow rate of 3000 nL/min. The trap column was then switched in line with the ProntoSIL C18AQ analytical column (100 µm, ID × 150 mm, 3 micron 200 Å). The reversed phase elution gradient was from 2% to 20% to 45% B over 60 min, total 85 min at a flow rate of 1000 nL/min. Solvent A was LCMS-grade water with 0.1% formic acid; solvent B was LCMS-grade ACN with 0.1% FA. The mass spectrometer was set up in positive ionization MS mode with a mass range of *m*/*z* 130–2200. All samples (including biological repeats) were analysed in duplicate. To identify the peptides of interest, a pool from each treatment group was made by combining 5 µL of every sample digest and duplicates were run on the Impact HD mass spectrometer. More specifically, the pooled samples were measured in data-dependent MS/MS mode, where the acquisition speed was 2 Hz in MS and 1–20 Hz in MS/MS mode depending on precursor intensity. Ten precursors were selected in the *m*/*z* range of 350–1200, with preference for doubly or triply charged peptides. The analysis was performed in positive ionization mode with a dynamic exclusion of 60 s.

#### 3.4.3. Protein Identification and Quantification

Following LC-MS/MS analysis, the QTOF data were searched using the Peaks Studio 8.5 search algorithm (Bioinformatics Solutions, Waterloo, ON, Canada) against an in-house *Mytilus* sequence database and the uniprot-mollusca-200718-290-109 sequence database. The following parameters were used: A precursor mass tolerance of 0.15 Da and a fragment mass tolerance of 0.15 Da were allowed, trypsin was specified as the digestive enzyme, and up to 2 missed cleavages were allowed. Carbamidomethyl (C) was specified as a fixed modification, and oxidation (M) and deamidation (NQ) were chosen as variable modifications. A false discovery rate (FDR) of 1% was used for peptide identification in Peaks. In addition, the Peptide Hit Threshold (−10log_) was set to 30, de novo only 15% of ALC, and only proteins with a minimum of 1 unique peptide identification were included.

### 3.5. Data Analysis

One-way analysis of variance (ANOVA) followed by a Dunnett test against the DMSO control (*p* < 0.01) was used to analyse antisettlement, antibacterial and antimicroalgal screening data as well as *Artemia salina* ecotoxicity data. The semi-maximum response concentration that inhibited 50% of mussel larval settlement (EC_50_), response concentration that inhibited bacterial growth (IC) and the median lethal dose (LC_50_) were assessed using Probit regression analysis, when applicable. Pearson Goodness-of-fit (Chi-Square) significance was considered at *p* < 0.01 for this analysis, and a 95% lower confidence limit (LCL) and a 95% upper confidence limit (UCL) were presented. In proteomic analysis, the mixed model ANOVA was utilized to report protein abundance differences between treatments at a confidence level of 95%. The mixed model ANOVA was performed on log10(*x* + 1)-transformed values from 4 biological replicates. Unless otherwise stated, the software IBM SPSS Statistics (version 21, SPSS Inc., Chicago, IL, USA) was used for statistical analysis.

## 4. Conclusions

This study elucidates the potential of portoamides as new eco-friendly AF agents, given their high effectiveness in the prevention of the attachment of mussel larvae, enabling the reversibility of the effect after exposure, and their antibacterial properties towards marine biofilm-forming bacteria, while lacking the toxicity towards both target and non-target species. This AF potential is even further enhanced by the fact that the major molecular target of portoamides, in the important macrofouling species *Mytilus galloprovincialis,* is the failure in ATPase proton-transporting activity, and thus portoamides could be promising as an effective way of controlling the attachment of a wide range of biofouling species.

## Figures and Tables

**Table 1:** Bacterial growth inhibition activitiy of portoamides.

Compounds	EC_30_ (μM)		
Portoamides	*Cobetia marina*	*Halomonas aquamarina*	*Pseudoalteromonas atlantica*
	14.56 (95% CI: 10.21–23.75)	14.83 (95% CI: 10.41–24.66)	13.59 (95% CI: 9.67–21.45)

EC_30_, minimum concentration that inhibited 30% of bacterial growth. CI, confidence interval.

**Table 2:** Differentially expressed proteins (*p*-value < 0.05) in *Mytilus galloprovincialis* larvae exposed to portoamides (16 μM). Besides the mean log10-transformed abundance values, the names of the identified proteins and the main metabolic processes involved are also listed in the table. Protein accession numbers are from NCBI.

	Mean of log10 (*x* + 1)-Transformed Values		*p*-Value	Accession	Protein Name	Metabolic Process
	Control	Portoamides				
**Energy Metabolism**	2.465	0.304	0.004	XP_009028970.1	ATP synthase subunit d, mitochondrial	ATP synthesis
	3.742	3.335	0.011	XP_021356377.1	ATP synthase subunit beta mitochondrial	
	3.783	3.494	0.012	ABJ51956.1	H+ ATPase a subunit mitochondrial	
	3.424	3.059	0.018	XP_009064140.1	ATP synthase subunit gamma	
	3.455	2.890	0.018	AFI56365.1	Isocitrate dehydrogenase	Tricarboxylic Acid cycle
	3.401	3.023	0.027	AAF27650.1	Malate dehydrogenase	
	2.825	2.405	0.031	XP_022314664.1	Glutamate dehydrogenase mitochondrial	
**Structural**	4.116	3.771	0.050	XP_022317649.1	Myosin heavy chain striated muscle	Muscle structural protein; contractionmovement
	3.855	3.469	0.046	CAB64663.1	Pedal retractor muscle myosin heavy chain	
	4.386	3.882	0.050	XP_014664190.1	Tubulin beta chain	Cytoskeleton constituent
	3.857	3.379	0.038	XP_021370666.1	Tubulin alpha-1A chain	
	3.166	2.623	0.035	XP_022332848.1	Cilia- and flagella-associated protein	
	3.756	3.064	0.021	KFB49247.1	Myosin heavy chain	
	3.149	2.888	0.018	NP_001292292.1	Tubulin beta chain	
	3.497	2.929	0.015	XP_009029528.1	Tubulin beta chain	
	3.269	2.819	0.004	XP_021350592.1	Myosin heavy chain	
	2.660	1.971	0.024	XP_011416098.1	Tektin-4	Structural, cell motility
	2.440	1.054	0.027	XP_021370563.1	Radial spoke head protein 4	
	2.343	1.106	0.031	XP_021363337.1	Radial spoke head protein 9	
	3.31471	2.727	0.031	XP_011450983.1	Tektin-3	
	1.974	0	0.037	XP_021375365.1	Collagen, type VI, alpha 3	Extracellular matrix organization
**Gene Transcription/Translation**	2.990	2.673	0.049	XP_021341263.1	Dolichyl-diphosphooligosaccharide protein glycosyltransferase	Protein modification, PTMs
	4.011	3.579	0.040	AKS48144.1	Arginine kinase	
	3.020	2.360	0.016	XP_021367901.1	Dolichyl-diphosphooligosaccharide protein glycosyltransferase 48 kDa subunit	
	3.854	3.596	0.048	XP_018019106.1	14-3-3 protein	Chaperone, protein activity stabilization/regulation
	3.474	2.958	0.044	XP_011444269.1	Peptidyl-prolyl cis-trans isomerase	
	3.578	3.200	0.041	CAJ85741.1	Heat shock protein 90	
	2.515	2.191	0.038	XP_011446200.1	Protein disulfide-isomerase A6	
	2.431	2.025	0.019	Q6WV66.3	Histone H2A	Chromatin function, gene transcription
	2.493	2.080	0.027	XP_021377317.1	Histone H2A	
	3.618	3.309	0.035	XP_021363857.1	Leucine-rich repeat flightless-interacting protein 2	
	4.961	4.649	0.042	XP_014590042.1	Histone H4	
	1.968	0	0.024	XP_012945445.1	Heterogeneous nuclear ribonucleoprotein 87F	Ribossome constituent/function, protein translation
	2.738	2.356	0.029	XP_021369622.1	60S ribosomal protein L7a	
	3.071	2.707	0.036	XP_022290789.1	40S ribosomal protein S4	
	3.332	2.965	0.041	XP_011438308.1	40S ribosomal protein S3	
	3.050	2.675	0.042	XP_011441368.1	40S ribosomal protein S13	
	3.250	2.727	0.043	XP_021370373.1	40S ribosomal protein S25	
	3.292	3.038	0.048	XP_013068480.1	60S ribosomal protein L11	
**Transport**	1.482	0	0.043	XP_021354511.1	Clathrin heavy chain 1	Vesicle mediated transport
	2.565	2.174	0.011	XP_022317867.1	Annexin B9	
	3.565	3.113	0.038	SCN46548.1	ADP, ATP carrier protein	Transporters, carrier proteins
	3.667	3.260	0.046	ADI56517.1	Voltage-dependent anion channel	
**Other Processes**	2.595	2.247	0.0468	XP_022343934.1	6-phosphogluconate dehydrogenase decarboxylating	Synthesis of NADPH and 5-carbon sugar precursor
	3.284	2.942	0.042	AFQ35984.1	Glutathione S-transferase sigma 2	Detoxification, response to oxidative stress

Note: See Supplementary Table S1 for a summary of LC-MS/MS protein identification results.

## Supplementary Materials

The following are available online at http://www.mdpi.com/1660-3397/17/2/111/s1, Table S1: Summary of LC-MS/MS protein identification results. Figure S1: Relative proportions of portoamides A and B. Absorption spectra (**A**) obtained by the analytical method with the absorbance as a function of time. The first peak represents portoamide A, while the second peak is portoamide B. The PDA spectrum (**B**), for each absorbance spectrum, with absorbance at the wavelength of 276.0 nm.
