# Supplementary material for: A Multi-Bioassay Integrated Approach to Assess the Antifouling Potential of the Cyanobacterial Metabolites Portoamides
Source: Mar Drugs. 2019 Feb 12;17(2):111. doi: 10.3390/md17020111 (PMC6410096; doi:10.3390/md17020111)
Supplement: Supplementary file 1 [file marinedrugs-17-00111-s001.pdf]

marine drugs

Supplementary Materials

A Multi-Bioassay Integrated Approach to Assess the Antifouling Potential of the Cyanobacterial Metabolites Portoamides

Jorge Antunes [1,2], Sandra Pereira [1], Tiago Ribeiro [1], Jeffrey E. Plowman [3], Ancy Thomas [3], Stefan Clerens [3,4,5,] Alexandre Campos [1], Vitor Vasconcelos [1,2,*] and Joana R. Almeida [1,*]

[1] Centro Interdisciplinar de Investigação Marinha e Ambiental (CIIMAR), Universidade do Porto, Terminal de Cruzeiros do Porto de Leixões, Av. General Norton de Matos s/n 4450-208 Matosinhos, Portugal; jorgetantunes@gmail.com (J.A.), sandra.c.pereira28@gmail.com (S.P.); tiago.amribeiro8@gmail.com (T.R.); acampos@ciimar.up.pt (A.C.)

[2] Departamento de Biologia, Faculdade de Ciências, Universidade do Porto, Rua do Campo Alegre, P 4069-007 Porto, Portugal

[3] AgResearch Ltd., 1365 Springs Rd, Lincoln 7674, New Zealand; Jeff.Plowman@agresearch.co.nz (J.E.P.); Ancy.Thomas@agresearch.co.z (A.T.); Stefan.Clerens@agresearch.co.nz (S.C.)

[4] Biomolecular Interaction Centre, University of Canterbury, Christchurch P 8140, New Zealand

[5] Riddet Institute, Massey University, Palmerston North P 4442 New Zealand

* Correspondence: vmvascon@fc.up.pt (V.V.); jalmeida@ciimar.up.pt (J.R.A.)

List of Supplementary Materials

Table S1: Summary of LC-MS/MS protein identification results.

Figure S1: Structures and relative proportions of portoamides A and B.

marine drugs

MDPI

Table S1. Summary of LC-MS/MS protein Identification results. The QTOF data from the Impact HD mass spectrometer equipped with a CaptiveSpray source (Bruker Daltonik, Bremen, Germany) were searched using the Peaks Studio 8.5 search algorithm (Bioinformatics Solutions, Waterloo, ON, Canada).

Protein Name	Accession	Significance	Coverage (%)	#Peptides	#Unique Peptides	PTM	Avg. Mass
**Energy Metabolism**							
ATP synthase subunit d, mitochondrial	XP_009028970.1	14.86	7	1	1	N	20040
ATP synthase subunit beta mitochondrial	XP_021356377.1	57.57	30	13	13	Y	56361
H+ ATPase a subunit mitochondrial	ABJ51956.1	151.76	22	12	12	N	59490<br>59490
ATP synthase subunit gamma	XP_009064140.1	68.99	11	3	3	N	32306
Isocitrate dehydrogenase	AFI56365.1	32.31	12	5	5	N	50501
Malate dehydrogenase	AAF27650.1	25.87	7	2	2	N	35955
Glutamate dehydrogenase mitochondrial	XP_022314664.1	8.96	2	1	1	N	60325
**Structural**							
Myosin heavy chain striated muscle	XP_022317649.1	200	25	47	41	Y	228093
Pedal retractor muscle	CAB64663.1	153.53	33	21	19	N	87460
Tubulin beta chain	XP_014664190.1	200	28	9	5	Y	49949
Tubulin alpha-1A chain	XP_021370666.1	50.58	39	13	2	Y	38578
Cilia- and flagella-associated protein	XP_022332848.1	23.01	8	2	2	N	33218
Myosin heavy chain	KFB49247.1	139.38	39	6	5	Y	20888
Tubulin beta chain	NP_001292292.1	37.69	23	6	2	Y	33218
Tubulin beta chain	XP_009029528.1	9.32	30	5	1	N	27930

Myosin heavy chain	XP_021350592.1	65.88	5	8	8	Y	230478
Tektin-4	XP_011416098.1	24.6	6	2	2	N	52138
Radial spoke head protein 4	XP_021370563.1	8.73	4	1	1	N	50579
Radial spoke head protein 9	XP_021363337.1	8.56	4	1	1	N	36000
Tektin-3	XP_011450983.1	49.81	13	7	7	Y	62040
Collagen, type VI, alpha 3	XP_021375365.1	18.34	7	1	1	N	15965
**Protein Activity Regulation**							
Dolichyl-diphosphooligosacc-haride protein glycosyltransferase	XP_021341263.1	27.49	8	4	4	N	68234
Arginine kinase	AKS48144.1	132.06	20	6	6	Y	42147
Dolichyl-diphosphooligosacc-haride protein glycosyltransferase 48 kDa subunit	XP_021367901.1	22.72	4	2	2	N	48797
14-3-3 protein	XP_018019106.1	200	26	6	4	Y	29977
Peptidyl-prolyl cis-trans isomerase	XP_011444269.1	26.14	9	2	2	N	29564
heat shock protein 90	CAJ85741.1	82.08	12	8	8	N	83125
Protein disulfide-isomerase A6	XP_011446200.1	8.85	2	1	1	N	47641
**Gene Transcription/Translation**							
Histone H2A	Q6WV66.3	8.82	37	4	1	N	13360
Histone H2A	XP_021377317.1	8.79	34	4	1	Y	14212
Leucine-rich repeat flightless-interacting protein 2	XP_021363857.1	9.33	3	1	1	N	45577
Histone H4	XP_014590042.1	200	50	6	6	Y	11367
Heterogeneous nuclear ribonucleoprotein 87F	XP_012945445.1	18.28	3	1	1	N	36578

60S ribosomal protein L7a	XP_021369622.1	8.9	5	1	1	N	30513
40S ribosomal protein S4	XP_022290789.1	24.47	7	2	2	N	29583
40S ribosomal protein S3	XP_011438308.1	33.34	9	2	2	N	26632
40S ribosomal protein S13	XP_011441368.1	8.94	8	1	1	N	17356
40S ribosomal protein S25	XP_021370373.1	27.7	18	2	2	N	16837
60S ribosomal protein L11	XP_013068480.1	30.49	13	2	2	N	20256
**Transport**							
Clathrin heavy chain 1	XP_021354511.1	11.55	1	1	1	N	192108
Annexin B9	XP_022317867.1	8.76	5	1	1	N	35854
ADP, ATP carrier protein	SCN46548.1	77.98	15	4	4	Y	33267
Voltage-dependent anion channel	ADI56517.1	75.71	12	3	3	N	30686
**Other processes**							
6-phosphogluconate dehydrogenase decarboxylating	XP_022343934.1	8.76	2	1	1	N	53103
Glutathione S-transferase sigma 2	AFQ35984.1	22.18	11	2	2	N	23359

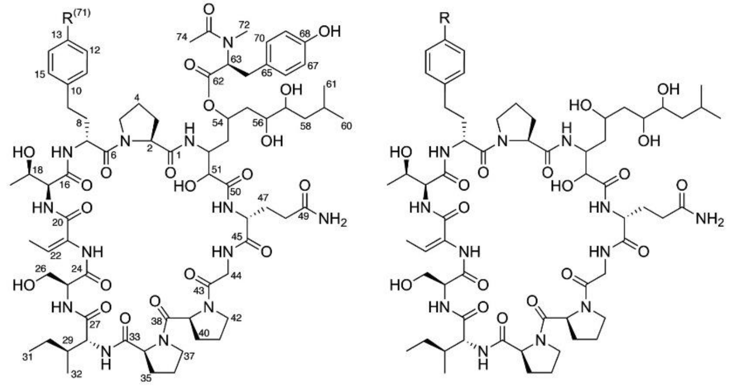

1: R = OCH_3
2: R = H

3: R = OCH_3
4: R = H

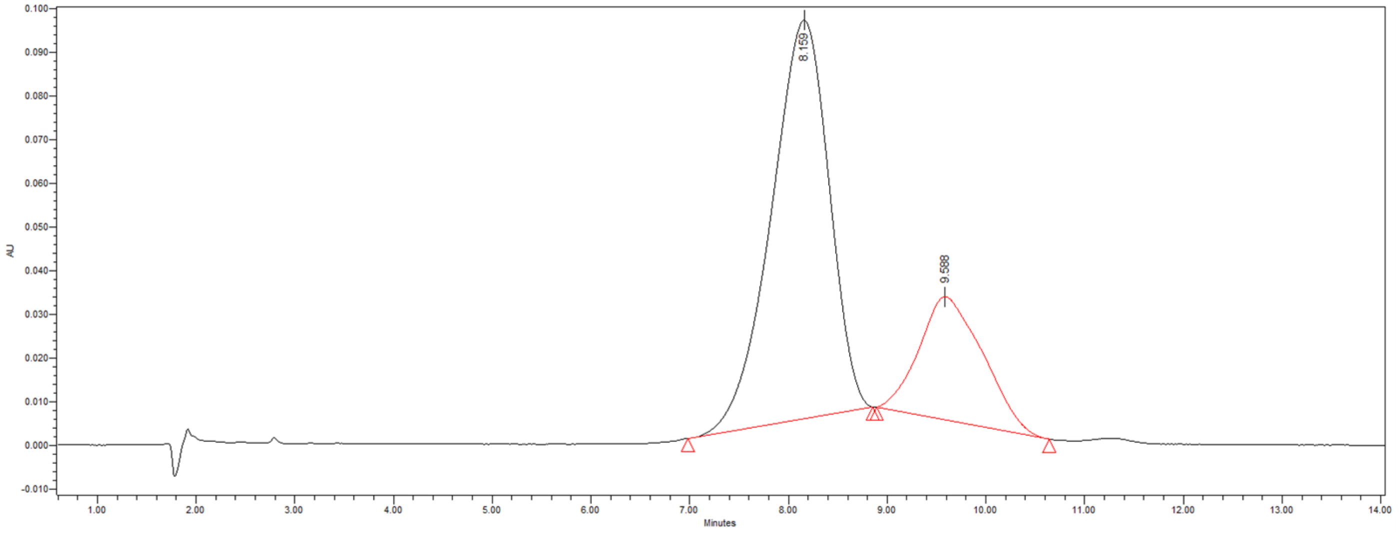

Figure S1. Absorption spectra (**A**) obtained by the analytic method with the absorbance as function of time. The first peak represents portoamide A, while the second peak is portoamide B. The PDA spectrum (**B**), for each absorbance spectrum, with absorbance in the wavelength of 276.0 nm. Structures as represented by Leão et al. [25].
